# Thermal Energy Transfer between Helium Gas and Graphene Surface According to Molecular Dynamics Simulations and the Monte Carlo Method

**DOI:** 10.3390/nano12162855

**Published:** 2022-08-18

**Authors:** Lin Zhang, Heng Ban

**Affiliations:** 1 Department of Engineering Mechanics, School of Civil Engineering, Shandong University, Jinan 250061, China; 2 Department of Mechanical Engineering and Materials Science, University of Pittsburgh, Pittsburgh, PA 15261, USA

**Keywords:** thermal accommodation, gas–surface interaction, graphene, molecular dynamics, Monte Carlo method

## Abstract

The scattering of gases on solid surfaces plays a vital role in many advanced technologies. In this study, the scattering behavior of helium on graphene surfaces was investigated, including the thermal accommodation coefficient (TAC), outgoing zenith angle of helium, bounce number, and interaction time. First, we performed molecular dynamics simulations to describe the incident angle-resolved behaviors, and showed that the scattering is highly dependent on the zenith angle of incident helium but insensitive to the azimuthal angle. The contribution of the normal velocity component of the incident helium dominated the energy transfer. The nonlinear relationship of the parameters to the zenith angle of the incident helium could be suppressed by increasing the graphene temperature or decreasing the speed of the incident helium. Subsequently, the scattering performance considering all gas molecules in the hemispherical space was evaluated using the Monte Carlo method with angle-resolved results. The result showed that the TAC, its nominal components, and the zenith angle of the scattered helium increased with higher speeds of incident helium and lower temperatures of graphene. This study should provide a fundamental understanding of energy transfer between gas and two-dimensional materials and guidelines to tune the scattering behavior between them.

## 1. Introduction

The scattering dynamics of gas-phase molecules on the surface of solids [1,2,3] play an essential role in many technologies for interfacial energy and momentum accommodation, and affect the efficiency of energy and mass transport, the friction at the interface, and the rate of catalysis and corrosion [4,5,6,7,8,9]. The influence of gas–surface scattering becomes especially significant for non-continuum flows characterized by a high Knudsen number, defined as the ratio of the mean free path length of gas molecules to the characteristic length of the system. One typical scenario involves structures of a small characteristic length, such as a microchannel [10] or nanochannel environment [11,12,13], as well as gas–molecular structure interfaces [14,15,16]. Microchannels are an integral part of micro-electro-mechanical devices and high-precision equipment [11,12]. Gas surface interaction significantly affects the heat transfer performance in microchannel cooling applications [17,18], the gap conductance between fuel and cladding in nuclear engineering [19], and the adsorption/desorption of gas molecules in solid-state gas sensors. The scattering dynamics of the gas on a solid surface play a vital role in these applications. Hence, there is a universal need to elucidate the energy transport mechanism at gas–surface interfaces.

The energy transfer at the gas–surface interface is usually evaluated by the parameter of the thermal accommodation coefficient (TAC) [20], which describes the ensemble behavior of the gas molecules on a solid surface for engineering applications. In typical experimental systems, heat flux is generated from an electrically heated filament to a coaxial cylinder immersed in a bath at a constant temperature [21,22] or between two parallel plates at a controlled temperature [23,24]. Then, the TAC value is calculated using the measured heat flux and temperature distribution, as described by different theoretical models [21,25], such as the low-pressure method (free molecular method) [22,26], the temperature-jump method [27], and the mean free path method [28]. For numerical methods, the molecular dynamics (MD) method has been widely used to understand gas–surface scattering and calculate the thermal accommodation coefficient. The MD method can provide more details, such as complicated gas–surface atomistic interactions, than other techniques [29,30]. Similar to experimental setups, MD systems can consist of atoms of a solid surface and numerous gas molecules. In this way, the system can explicitly describe the collective behavior of gas molecules. For instance, the TAC values of helium gases scattered upon nanoscale particles of Ar and N_2_ were found to be independent of the gas pressure and temperature [31]. MD simulations of monatomic, diatomic, and mixed gas molecules show that the TAC is dependent on gas–surface interaction strength, gas–solid mass ratio, surface adsorption, surface curvature, and solid stiffness [32,33,34,35]. It is worth noting that the gas molecules of these reported systems are at very high pressures to dramatically reduce the computation time with more frequent scattering than the normal condition. However, those systems would suffer from low computational efficiency in cases of free molecular flow, and the calculated value combines the gas–gas scattering effect near the surface for gas molecules of high density.

Instead, the molecular beam method provides a unique angle to investigate gas–surface scattering behavior [36,37,38,39,40,41,42,43]. In an experimental setup, velocity selectors along with detectors are used to build a statistical correlation of the parameters, such as the dependency of the speeds and angles between the incident and scattered gas molecules. Besides thermal scattering, hyperthermal scattering, where the energy of an incident molecule is 1~15 eV, can also be studied by heating the gas molecules to above 1000 °C with molecular beam systems [44,45,46,47]. However, experimental studies with the molecular beam method can only focus on incident zenith angles from 30° to 70° due to equipment limitations [36]. The understanding of gas–surface scattering is lacking when the incident angle is smaller than 30° or greater than 70°. On the other hand, the gas molecules can have any given incident angle in the MD system, elucidating the scattering mechanism [36], and resolving the contributions by normal and planar accommodation. Most previous studies focused on bulk materials such as metal and graphite crystals [36,48,49]. Few studies reported on the thermal energy transfer between gas and two-dimensional (2D) materials. The studies of gas molecules or ions with two-dimensional materials are more related to defects and damage to the 2D material due to high-energy gas molecules or ions [50,51,52]. It is still unclear how gas–2D surface interaction affects thermal energy transport.

Here, we provide a comprehensive analysis of the scattering behavior of helium atoms on monolayer graphene using both the incident-angle resolved evaluation and integrated performance considering gas molecules from the hemispherical space. We performed MD simulations to model the molecular beam scattering of helium atoms on monolayer graphene and analyzed the zenith and azimuthal angle effects of the incident helium on the interfacial energy transfer. The contribution of the normal and in-plane velocity components of helium to energy transfer was evaluated using both the normal and tangential thermal accommodation coefficients. In MD simulations, graphene is controlled at a relatively high temperature for a particular application in an extreme environment, such as nuclear engineering [53] or soot particle [54] evaluation by thermal property analysis [55]. The effects of temperature of the graphene surface and incident helium on scattering behaviors were further studied. Then, we applied Monte Carlo simulations to evaluate the relative frequencies of the specified zenith-angle ranges of the incident helium. The integrated performance considering gas molecules from the hemispherical space was predicted by weighting the parameters based on the zenith angles of incident helium according to relative frequencies. The scattering behavior of helium on graphene was elucidated and correlated by the resolution of incident angles and integrated evaluations. This study should enrich the understanding of gas–surface energy transfer and provide new angles to tune free molecular flows with surfaces or channels of 2D materials.

## 2. Computational Methods

### 2.1. Atomistic Models

**Figure 1:**
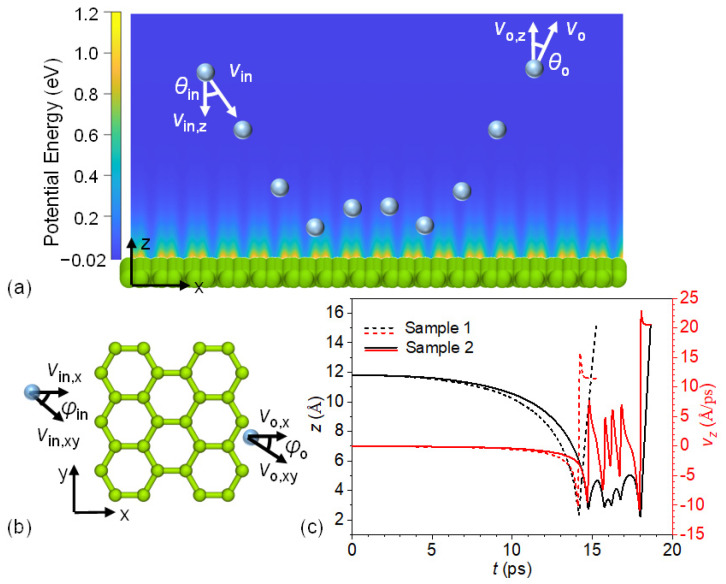
(**a**) An illustration of a sampling system composed of a helium atom (blue sphere) and monolayer graphene (green sphere layer). The multiple blue spheres illustrate one trajectory of a helium atom scattered on the graphene surface. The background shows the potential energy of the system relative to the case where the helium atom is 20 Å from the graphene surface. θ and V stand for the polar angle and the velocity of helium. The subscripts “in” and “o” denote the incident and scattered helium, respectively. (**b**) Top view of the helium–graphene system. ϕ denotes the azimuth angle, and the subscript “xy” represents the in-plane component. (**c**) Two characteristic examples of the scattering process of helium on monolayer graphene. The black curves are the distances of helium atoms from the top surface of the graphene, and the red curves are the normal velocity components of helium.

As shown in Figure 1a,b, the sampling system for molecular dynamics (MD) simulations was composed of a helium gas atom (blue sphere) and monolayer graphene (a layer of green spheres). The bond length of the graphene was initialized as 1.42 Å. Periodic boundary conditions were applied in all three directions. The size of the system was 63.915 Å in the x direction and 61.5 Å in the y direction. To evaluate the system size effect, we built and investigated a system with graphene of 127.83 Å and 123.0 Å in the x and y directions, respectively, as shown in Figure S1. We illustrated the fluctuations in the randomly selected carbon atoms in the NVE process and those of the closest carbon atoms to the helium atom during scattering for both systems. The results show that the fluctuations in the carbon atoms due to the scattering were within the range of the thermal fluctuations. The cumulative average values of TACs were comparable between the initial and enlarged systems, and the differences between them are within the scope of the error bars. Hence, the system with graphene dimensions of 63.915 Å and 61.5 Å was used to study the scattering behavior of helium on graphene.

A vacuum layer of 50 Å was added on each side of the graphene to avoid interaction between real helium atoms and the imaged carbon atoms of the graphene. The helium atom was initially placed at a plane 40 Å higher than the top surface of the graphene. The atomistic interactions of graphene were described using the modified AIREBO force field [56], which has been widely used for MD simulations of graphene. The interatomic potential of carbon atoms of graphene and helium was expressed as *E* = 4 × ε × [(σ/r)^12^ − (σ/r)^6^] with a cutoff of 12.0 Å, where ε takes 1.6228 meV, and σ is 3.053 Å. Those values come from the OPLS-AA force field [57] according to the Lorentz–Berthelot mixing rules. 

### 2.2. Molecular Dynamics Simulations

All molecular dynamics simulations were conducted with the LAMMPS code [58]. A helium atom was first fixed 40 Å from the top surface of the graphene in order to relax the graphene independently. The velocity Verlet integrator was used for time integration. Five steps were taken to prepare the graphene for the production run. First, the graphene was equilibrated for 5.0 ps in the NPT ensemble using a Nosé–Hoover thermostat with a time constant of 0.25 ps at a constant temperature of 300 K, and the simulation boxes in the x and y directions were adjusted independently via a Nosé–Hoover barostat with a time constant of 1.0 ps and zero in-plane pressure. Second, the graphene was heated to the temperature specified in the NPT ensemble at a rate of 10 K/ps. Then, the graphene was equilibrated at the elevated temperature in the NPT ensemble for 30.0 ps and the NVT ensemble for 5.0 ps. The time steps in the above simulations were 1.0 fs. Finally, the graphene was equilibrated in the NVE ensemble for 5.0 ps with a time step of 0.1 fs. At this point the graphene was well prepared for simulation of the scattering process with a helium atom at different sampled initial locations and incident velocities. The initial position of the helium atom was randomly generated by a uniform distribution at a plane 12.0 Å or 11.8 Å above the top surface of the graphene. The value of 11.8 was applied when the initial speed of helium was smaller than 0.1 Å/ps, which located the helium within the interaction zone of graphene. The initial velocity of the helium atom was designated by the root mean square velocity of the molecule at the given temperature according to the Maxwell–Boltzmann distribution. It is described by $\sqrt{3k_{B}T/m}$ where *k_B_* is the Boltzmann constant, *T* is the thermodynamic temperature, and *m* is the mass of the helium atom. The incident zenith angle (polar angle) and azimuthal angle of helium were specified to evaluate the effect of the incident angle of the molecular beam. It is worth noting that samples with a polar angle of 90° correspond to the cases where the normal velocity component is zero. The helium atom could be attracted to the graphene surface purely by the van der Waals interactions. During the scattering of helium on graphene, helium gained kinetic energy from the graphene and eventually escaped from the attraction of the graphene surface. The simulation ceased when the outgoing helium was 15.0 Å from the graphene surface. The velocity and coordinates of the helium atom were recorded during the scattering process for further analysis.

### 2.3. Thermal Accommodation Coefficient Calculation

The Maxwell model has been widely applied for calculation of the thermal accommodation coefficient (TAC, α). This model indicates that the α fraction of gas molecules has diffuse scattering on the solid surface, and the remaining fraction has specular scattering. In this way, TAC is described as

(1) $$\alpha =\frac{\langle E_{o}\rangle -\langle E_{in}\rangle }{\langle E_{o}(T_{s})\rangle -\langle E_{in}\rangle }=\frac{\langle \frac{1}{2}mv_{o}^{2}\rangle -\frac{1}{2}mv_{in}^{2}}{2k_{B}T_{s}-\frac{3}{2}k_{B}T_{g}}$$

where *E*, *v*, *m*, and *T* denote the kinetic energy, speed, mass, and thermodynamic temperature of the helium atom, respectively. *T_s_* and *T_g_* indicate the temperature of the solid surface and the incident helium gas. *k_B_* is the Boltzmann constant. The subscripts “*in*” and “*o*” indicate the incident and scattered helium, respectively.

To identify the contributions of the in-plane and out-of-plane components of the energy transfer, we decomposed the thermal energy accommodation into the nominal normal thermal accommodation coefficient (NNTAC, *α_n_*) and nominal tangential thermal accommodation coefficient (NTTAC, *α_τ_*) by

(2) $$\alpha_{n}=\frac{\frac{1}{2}mv_{o,z}^{2}-\frac{1}{2}mv_{in,z}^{2}}{2k_{B}T_{s}-\frac{3}{2}k_{B}T_{g}}$$

(3) $$\alpha_{\tau }=\frac{\frac{1}{2}mv_{o,xy}^{2}-\frac{1}{2}mv_{in,xy}^{2}}{2k_{B}T_{s}-\frac{3}{2}k_{B}T_{g}}$$

(4) $$\alpha =\alpha_{n}+\alpha_{\tau }$$

where *v_in,xy_* and *v_in,z_* denote the in-plane and normal components of the speed of the helium atom, respectively.

According to the definition of TAC, the real normal thermal accommodation coefficient (RNTAC, α_n_^*^) and real tangential thermal accommodation coefficient (RTTAC, α_τ_^*^) may also be evaluated using the following definition, given the zenith angle *θ_in_* of the incident helium:

(5) $$\alpha_{n}^{*}=\frac{\langle E_{o,n}\rangle -\langle E_{in,n}\rangle }{\langle E_{o,n}(T_{s})\rangle -\langle E_{in,n}\rangle }=\frac{\frac{1}{2}mv_{o,z}^{2}-\frac{1}{2}mv_{in}^{2}\cos^{2}\theta_{in}}{k_{B}T_{s}-\frac{1}{2}mv_{in}^{2}\cos^{2}\theta_{in}}$$

(6) $$\alpha_{\tau }^{*}=\frac{\langle E_{o,\tau }\rangle -\langle E_{in,\tau }\rangle }{\langle E_{o,\tau }(T_{s})\rangle -\langle E_{in,\tau }\rangle }=\frac{\frac{1}{2}mv_{o,xy}^{2}-\frac{1}{2}mv_{in}^{2}\sin^{2}\theta_{in}}{k_{B}T_{s}-\frac{1}{2}mv_{in}^{2}\sin^{2}\theta_{in}}$$

### 2.4. Conversion of TAC of the Angle-Resolved Molecular Beam to TAC of the Hemispherical Molecular Beam

The angle-resolved TAC can be calculated by fixing the incident angle of the helium gas. In this study, the Monte Carlo Integration method was applied to convert the angle-resolved TAC into the TAC of all gas molecules from the hemispherical space. The probability density functions of gas velocity components with the fast atom effect are given by: [49]

(7) $$f_{i}(v_{i})=\frac{1}{\sqrt{2\pi }\sigma }\exp\left[-\frac{v_{i}^{2}}{2\sigma^{2}}\right]\;v_{i}\in (-\infty ,+\infty );\;i=x,y;\sigma =\sqrt{\frac{k_{B}T}{m}}$$

(8) $$f_{z}^{}(v_{z})=\frac{-v_{z}}{\sigma^{2}}\exp\left[-\frac{v_{z}^{2}}{2\sigma^{2}}\right]\;v_{z}\in (-\infty ,0]$$

(9) $$f_{v}(v)=\frac{v^{3}}{2}\frac{1}{\sigma^{4}}\exp\left[-\frac{v^{2}}{2\sigma^{2}}\right]\;v\in [0,\infty )$$

Then, the Monte Carlo method was used to sample the velocity components of the helium gas. In detail, the Box–Muller transform method with the acceptance–rejection method was used, such that *v_x_*, *v_y_*, *v_z,_* and *v* follow the distributions of Equations (7)–(9), respectively. In this way, the relative frequencies in the various ranges of the incident angles can be obtained. Subsequently, the angle-resolved TAC was recalculated by replacing the denominator of $2K_{B}T_{s}-\frac{3}{2}K_{B}T_{g}$ with $2K_{B}T_{s}-2K_{B}T_{g}$ to consider the fast atom effect of the diffusive scattering. Finally, the TAC of the semispherical gas molecules was calculated by integrating the angle-resolved TACs with the relative frequencies of the incident angles.

## 3. Results and Discussion

### 3.1. Scattering Process and Convergence Analyses

The scattering process of gas molecules on solid surfaces can be composed of the adsorption, trapping, and escaping stages. One of the latter two stages may not show up during the scattering. Figure 1c demonstrates two characteristic scattering processes of incident helium on the monolayer graphene surface at 1000 K. The speed, zenith angle, and azimuth angle of the incident helium of both samples are 17.652 Å/ps, 90°, and 0°, respectively. It is worth noting that the incident angle relative to the approaching local graphene surface may deviate slightly from the original values. In our simulation, we found that that was not significant. Here, we define the incident angles in a way consistent with the definition in the molecular beam experiments. The normal velocity component, *v_z_*, of incident helium was zero, such that the helium atom was adsorbed onto the graphene surface by the van der Waals interactions. As shown in Figure 1c, the distance of helium from the graphene surface and the normal velocity component of helium are depicted by the black and red curves, respectively. As the helium atom approached the graphene surface, *v_z_* decreased from zero to the negative maximum, where the negative sign indicates helium moving towards the graphene surface. During this process, helium atoms initially moved slowly downward and then accelerated. Afterward, the interaction between helium and graphene changed from attraction to repulsion, and the normal velocity component of helium declined. When helium arrived at the first minimum position above the graphene surface, *v_z_* became zero. After that, the helium atom bounced back from the graphene surface due to the repulsion forces between helium and graphene. The *v_z_* of helium atom increased quickly to the local maximum, where the attraction and repulsion between helium and graphene were balanced. Then, *v_z_* decreased as the attraction of graphene became weaker, dominating the graphene–helium interaction as helium moved away from the graphene. There were several different following behaviors. As shown in sample 1, the helium was far away enough from the graphene surface and had enough kinetic energy to escape from attraction by the graphene, leading to a scattering process with a single bounce. Another case is that the kinetic energy stored in the graphene might not be large enough for the first bounce-back process, and the helium atom would be attracted to the graphene surface again until it obtained enough kinetic energy to escape from attraction by the graphene. This is illustrated by sample 2, in which there were five bounces of helium until it flew away from the interaction region. In this study, we did not find any cases where the helium atom was trapped on the graphene surface within the simulation time.

The scattering behavior shown above varies randomly depending on the initial status of the graphene and the helium atom, such as the initial location, incident velocity, and incident angle of the helium atom. As shown in Figure S2, we found that the cumulative averages of TACs converged when 500 systems were sampled with the same incident velocities but random initial in-plane locations of the helium atom. For the illustrated samples in Figure S2, the incident speed of the helium atom was 17.652 Å/ps, which corresponds to the root mean square velocity of helium at 500 K; the zenith angle, θ, varied from 0° to 50° with an increment of 10°; and the azimuth angle was selected as 0° or 90°. The cumulative averages of the thermal accommodation coefficient (TAC), real normal thermal accommodation coefficient (RNTAC), real tangential thermal accommodation coefficient (RTTAC), and nominal normal thermal accommodation coefficient (NNTAC) converged with an increasing number of samples for all systems. For all data discussed below, the mean value and standard deviation were obtained via four evaluations with different initial velocity seeds of graphene, and each evaluation was calculated by averaging results from 500 samples with the same initial conditions except for the random in-plane initial position of the helium atom.

### 3.2. Effect of Zenith Angle of Incident Helium Atoms

**Figure 2:**
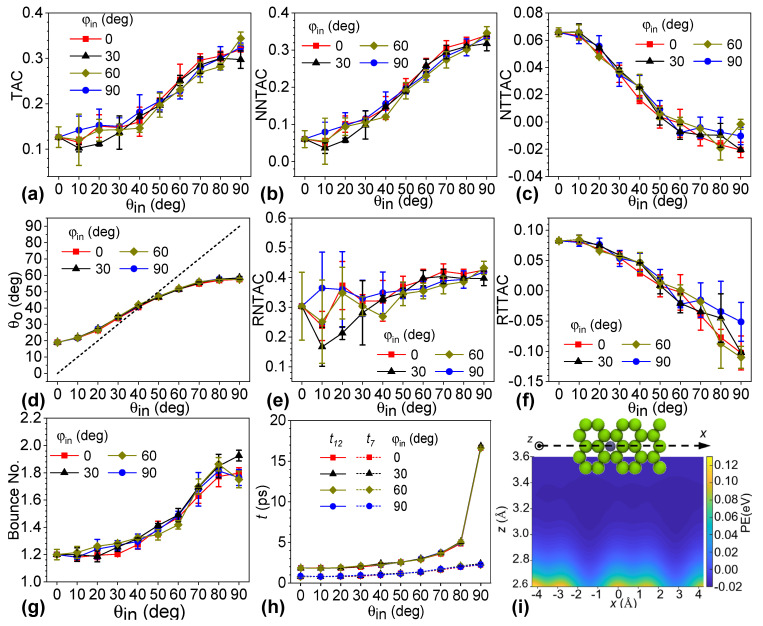
(**a**) Thermal accommodation coefficient (TAC), (**b**) nominal normal thermal accommodation coefficient (NNTAC), (**c**) nominal tangential thermal accommodation coefficient (NTTAC), (**d**) zenith angle of the scattered helium (*θ*_0_), (**e**) real normal thermal accommodation coefficient (RNTAC), (**f**) real tangential thermal accommodation coefficient (RTTAC), (**g**) bounce number of helium atoms, (**h**) total interaction time (t) versus zenith angle of the incident helium with the azimuth angle at 0°, 30°, 60°, and 90°, respectively. The subscripts 7 and 12 indicate distances between the helium and the surface to evaluate the collision time in units of angstroms. (**i**) Contour plot of relative potential energy when moving the helium in the denoted x–z plane.

Systems of monolayer graphene and a helium atom were simulated to elucidate the zenith-angle effect of the incident helium on the scattering process. The zenith angle (*θ_in_*) ranged from 0° to 90° with an increment of 10°, while the azimuthal angle (*φ_in_*) was specified at 0°, 30°, 60°, and 90°, respectively, as shown in Figure 2. Given *φ_in_*, TAC values increased significantly with larger values of *θ_in_*. Furthermore, TAC was insensitive to the *φ_in_* with the given *θ_in_*. For instance, TAC was 0.127 for a *θ_in_* of 0° and 0.319 for a *θ_in_* of 90°, which was 2.525 times that of the former value. For *θ_in_* of 0°, the incident velocity of the helium atom was normal to the graphene surface, and it was parallel to the surface for *θ_in_* of 90°. To evaluate the contribution of the normal and in-plane velocity components, we decomposed the TAC into nominal normal thermal accommodation (NNTAC) and nominal tangential thermal accommodation coefficients (NTTAC) in Equations (2) and (3). Figure 2b,c shows that NNTAC increased with an increasing zenith angle, while NTTAC decreased with a larger zenith angle. It is worthy of note that the value of NTTAC was much smaller than that of NNTAC. In particular, NTTAC was negative when *θ_in_* was greater than 60°, which means that in-plane kinetic energy was transferred from the helium to the graphene. As a result, we found that the kinetic energy component of the normal velocity contributed to a higher TAC and higher energy transfer along with a larger zenith angle. According to the definition in the Maxwell model, the real normal thermal accommodation coefficient (RNTAC, α_n_^*^) and real tangential thermal accommodation coefficient (RTTAC, α_τ_^*^) were calculated by Equations (5) and (6), respectively. Figure 2e,f shows that the trends of RNTAC and RTTAC were consistent with those of NNTAC and NTTAC, respectively. There were larger variances in RNTAC at small zenith angles and RTTAC at large zenith angles. The reason is that the normal kinetic energy of the incident helium atom was very close to that of the diffusively scattered helium atom, which resulted in a small value as the denominator and hence a larger variation in kinetic energy transferred in various systems. In detail, the normal kinetic energy of the helium atom after diffusive scattering (*k_B_*·*T_s_*) was 0.086 eV, and the kinetic energy of the incident helium atom at 500 K was 0.0646 eV, which was 75.1% of the former. When the zenith angle was smaller than 30°, the normal kinetic energy transferred by ideal diffusive scattering was a small value, which is the denominator. The case was the same when the zenith angle was larger than 70°. Interestingly, the variance in RNTAC at a small *θ_in_* was larger than that of RTTAC at a high value of *θ_in_*, which also demonstrates the importance of the normal velocity of the incident helium and the large contribution of the normal kinetic energy transfer during the scattering process.

A linear correlation between the zenith angles of incident and scattered gas molecules was previously reported for zenith angles in the range of 30°~70°. The zenith angles were limited due to restrictions in the experimental operations. In this study, the relationship was found to be nonlinear across the whole range of the incident angles of the helium atoms. As shown in Figure 2d, the curve deviated significantly from the dashed line as the zenith angle decreased or increased from this middle region between 30° and 70°. Here, the dashed line denotes that the zenith angles of the incidence were the same as those of the scattered helium. The scattered helium atom escaped from the graphene surface at a zenith angle of 19.03° when the incident helium was normal to the graphene. The in-plane force component applied to the helium atom could not be balanced due to the random vibration of carbon atoms of graphene, and the scattered helium atom deviated from the normal of the graphene surface depending on the attraction force. The zenith angle of the scattered helium was 57.84° for a *θ_in_* of 90°. In those cases, the normal velocity of the incident helium atoms was zero, and helium was adsorbed onto the graphene surface purely by the van der Waals interactions and gained kinetic energy normal to the surface during scattering. In-plane kinetic energy was transferred from the helium to the graphene, but the energy loss of helium was very low. The gain in normal kinetic energy was larger than the loss in in-plane kinetic energy, and the resultant energy transfer was enhanced. Moreover, the trends of zenith angles were consistent with that of the TAC and almost coincident with different azimuthal angles.

Figure 2g shows the bounce numbers of helium on graphene with different incident angles. The normal velocity of the outgoing helium atom determined whether the helium atom could escape from the surface. The curve presents three sections. First, the average bounce number increased slowly from 1.20 at a *θ_in_* of 0° until *θ_in_* reached 30°. For those cases, the normal velocities of helium were much larger than the in-plane velocities. After scattering, the helium atom could obtain both in-plane and normal kinetic energy and escape from the graphene surface. Second, the bounce number gradually increased with *θ_in_* above 30° until it reached 60°. In this range of *θ_in_*, the in-plane and normal velocity components of helium were comparable, the in-plane kinetic energy gain was marginal, and the major energy transfer was due to enhancement of the normal kinetic energy. Subsequently, the bounce number sharply increased with *θ_in_*, up to 1.82 at a *θ_in_* of 80°, and then decreased slightly from 80° to 90° except for the case of *φ_in_* of 30°. In this range, the in-plane velocity component of helium was larger than the normal component, and the in-plane energy transfer was from helium to graphene. Helium had to bounce more often on the graphene surface to gain enough energy to escape from the surface. The TAC averaged by the bounce number of helium increased from 0.105 to 0.177 as the zenith angle increased from 0° to 90°.

The interaction time between helium atoms and the graphene surface provides another angle to understand the scattering process of helium on the surface, as shown in Figure 2h. Interaction time was considered to start when the helium atom entered the cutoff distance above the graphene surface and to end when the scattered helium eventually returned beyond that distance. Curves of *t*_12_ denoting 12 Å above the graphene surface (the cutoff distance of the van der Waals interaction) were set as the threshold planes of the scattering. Those curves showed that, similarly to the bounce number, the interaction time had a correlation with the zenith angle of the incident helium except for the special cases of a *θ_in_* of 90°. The interaction time gradually increased from 1.839 ps to 4.968 ps for a *θ_in_* from 0° to 80°. The TAC averaged by the interaction time changed slightly. For *θ_in_* of 90°, the value rose sharply to 16.681 ps. To evaluate the influence of the normal velocity of the incident helium, we evaluated the interaction time by setting the threshold planes as 7 Å above the graphene surfaces. The dashed curves represent the results in Figure 2h. As shown in Figure 1c, the normal velocity of the incident helium atom changed significantly when the distance of the helium from the surface was less than 7 Å. By this definition, the interaction time increased with zenith angle, without the sharp jump. Helium atoms took a significantly longer time to accelerate, since the normal velocity of helium is zero with *θ_in_* of 90°, which decreased the efficiency of the heat energy transfer.

To understand why the zenith angle of incident helium significantly affected the TAC and scattering behaviors, we plotted the contour of the potential energy for the helium–graphene system. The potential energy was calculated by averaging the potential energy of the system in a dynamic run. First, a helium atom was introduced above the well-equilibrated monolayer graphene. Then, the potential energy was sampled by placing the helium atom in the x–z plane shown by an inlet with a resolution of 0.1Å. The reference potential energy was set when the helium atom was 20 Å above the graphene surface. Then, the system ran 1000 steps in the NVE ensemble with a timestep of 1.0 fs between each sample of the potential energy. In this way, the system was sampled 1000 times, and the averaged potential energies were plotted in the x–z plane in Figure 2i and in the y–z plane in Figure S3. It is worth noting that the location of the helium atom in the shown snapshot is not the origin point of the coordinate system. Both Figure 2i and Figure S3 show that the averaged potential energy was heterogeneous, and the gradient of the contour lines varied at different spots below the plane of 3.6 Å above the graphene surface. The variation is significant, especially in the region close to the surface. Hence, incident helium atoms of different zenith angles were subjected to various forces. Approaching the surface at different trajectories resulted in a significant change in the heat energy transfer.

### 3.3. Effect of Azimuthal Angle of Incident Helium Atoms

**Figure 3:**
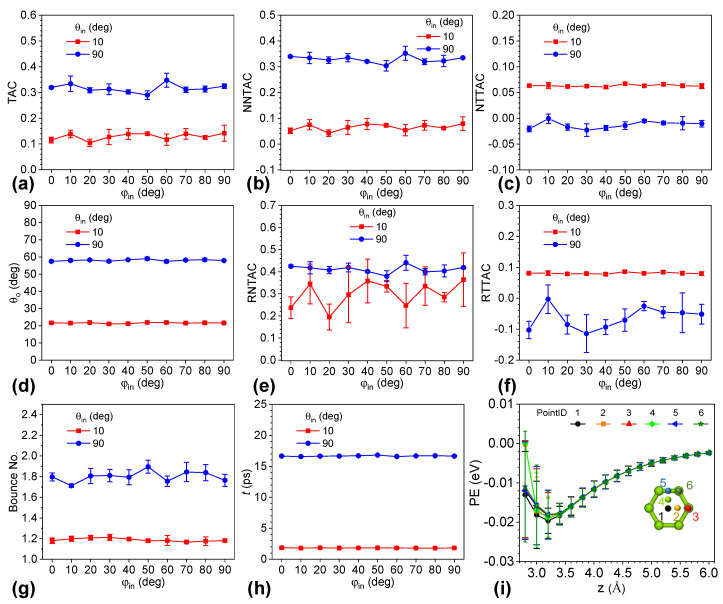
(**a**) TAC, (**b**) NNTAC, (**c**) NTTAC, (**d**) zenith angle of the scattered helium (*θ_o_*), (**e**) RNTAC, (**f**) RTTAC, (**g**) bounce number of helium, (**h**) total collision time versus azimuthal angle of the incident helium with the zenith angles at 0° and 90°, respectively. (**i**) Potential energy of the helium atom at six different in-plane locations versus distance of helium atom from the graphene surface. The inset indicates the six in-plane locations of helium atoms.

Figure 2 shows that the scattering process of helium on graphene was insensitive to the azimuths of 0°, 30°, 60°, and 90°. In order to better understand the effect of the azimuth of incident helium, we investigated the scattering behavior of helium atoms by increasing the azimuth from 0° to 90° at increments of 10°. The zenith angle was specified at 10° or 90° to consider two representative cases. The former represents a scenario where the helium atom is almost perpendicular to the graphene surface, while the latter is for a case where the incident velocity of the helium atom is parallel to the graphene surface. An angle of 10° was chosen instead of 0° because there was no difference in the in-plane velocity component of incident helium with a *θ_in_* of 0°. Figure 3a shows that the TAC values for a zenith angle of 10° varied from 0.104 to 0.142, with an average of 0.129 over the range of azimuthal angles. The TAC values of the zenith angle of 90° ranged from 0.290 to 0.347, with an average of 0.316. The variance in TACs was within 30% and 20% in the cases of *θ_in_* of 10° and 90°, respectively. Those results confirm that the energy transfer of helium on graphene was insensitive to the azimuthal angle. Figure 3b,c indicates that the effect of the azimuth was negligible for the normal and in-plane components of TAC. In the case of a *θ_in_* of 10°, the NNTAC values ranged from 0.043 to 0.079, while the NTTAC values fluctuated from 0.061 to 0.067. We found that the relative variance in the normal kinetic energy transfer was larger than that in the in-plane counterpart. The absolute values of NTTAC were comparable to those of the NNTAC. In the case of a *θ_in_* of 90°, the NNTAC values ranged from 0.304 to 0.353, while the NTTAC values fluctuated from −0.023 to −0.0006. It is notable that the in-plane energy transfers from helium to graphene and the change in NNTAC were much larger than those in NTTAC in absolute values. In terms of RNTAC and RTTAC, the red curve of RNTAC and blue cure of RTTAC show larger fluctuations in Figure 3e,f, which represent the cases of *θ_in_* of 10° and *θ_in_* of 90°, respectively. The reasons are the same as described above. On the other hand, we found that the variation in RNTAC with a *θ_in_* of 90˚ and RTTAC with a *θ_in_* of 10˚ was much lower than their counterparts, and the absolute values of those curves were much larger. We can conclude that energy transfer between helium and graphene is insensitive to the azimuth.

The zenith angle of scattered helium (*θ_o_*), average bounce number of helium, and interaction time of helium with graphene (*t*) were plotted with the various azimuthal angles in Figure 3d,g,h, respectively. The values of *θ_o_* for a *θ_in_* of 10° ranged between 21.056° and 21.932°, while the range of *θ_o_* for a *θ_in_* of 90° was from 57.463° to 59.042°. The variance in both cases was less than 4% and was much smaller than the change in TAC.

Regarding the parameter of the bounce number, we found that the values for a *θ_in_* of 10° varied between 1.167 and 1.212, and the difference was less than 4% of the lowest value. In the case of a *θ_in_* of 90°, the values lay between 1.713 and 1.896, and the change was within 10%. For interaction time, the range was from 1.803 ps to 1.867 ps in the case of a *θ_in_* of 10° and from 16.541 ps to 16.786 ps in the other case. Given these three parameters, the effect of the azimuthal angle was not significant and much smaller than the effect of the zenith angle, shown by the large differences between the red and blue curves.

To understand why the azimuth angle plays a negligible role in the scattering effect, we observed the potential energy of the helium–graphene system by fixing the helium at six in-plane locations, as shown by the inset of Figure 3i. The helium atom was placed 20 Å above the graphene surface, and the reference potential energy of the system was set. Then, we performed molecular dynamics simulations in the NVE ensemble for 1000 steps with a time step of 1.0 fs. Next, the potential energy of the system was sampled by placing the helium atom at the six in-plane locations and varying the distance between the helium and the graphene surface. In this way, the first loop was completed. Then, these steps were repeated until the potential energy was sampled for 1000 loops. Figure 3i shows the potential energy of the six in-plane locations with respect to the distance between the helium and the graphene. Given a plane parallel to the graphene surface, the potential-energy difference depends on the distance between the helium and the graphene surface. When the plane was much closer to the surface, we found that the average potential energy differed greatly. When the system was observed, the helium was placed at a fixed observation point, and the graphene dynamically vibrated during the dynamic run, which led to variance in the distance between them and hence a large range of potential energies. This variance was larger than the difference caused by the in-plane locations. As the distance of helium became further away from the graphene surface, both the variance from the dynamic vibration of graphene and that from the in-plane locations decreased. The former was more significant than the latter. In this way, the influence of dynamic vibration overwhelmed that of the in-plane location. As a result, the influence of the azimuth on the scattering process was less significant than random sampling, and can be negligible.

### 3.4. Effect of Temperature of Graphene Surfaces

**Figure 4:**
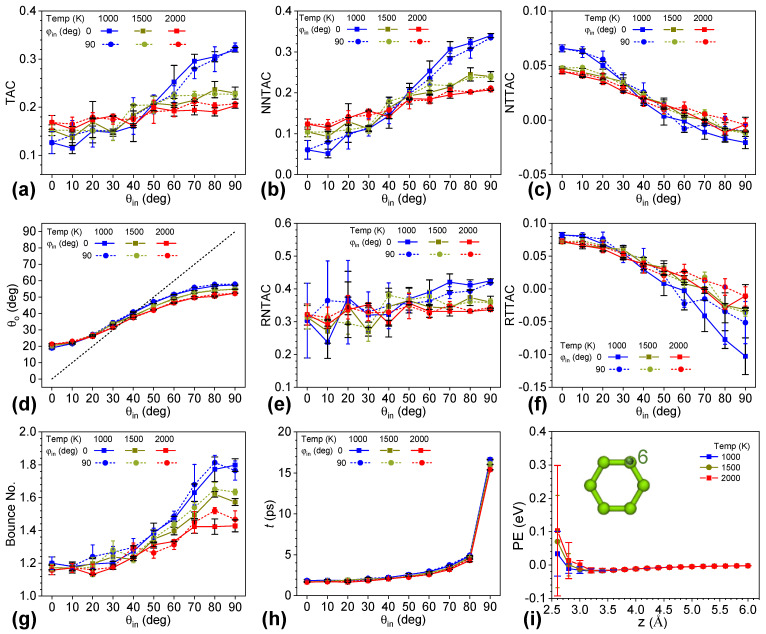
(**a**) TAC, (**b**) NNTAC, (**c**) NTTAC, (**d**) zenith angle of the scattered helium, (**e**) RNTAC, (**f**) RTTAC, (**g**) bounce number of helium, and (**h**) total collision time versus zenith angle of the incident helium with the azimuthal angle at 0° and 90°, respectively, and graphene temperatures of 1000 K, 1500 K, and 2000 K. The speed of helium corresponds to 500 K. (**i**) Potential energy of helium atom at the No. 6 in-plane location versus the distance of helium away from the graphene surface.

Here, we fixed the speed of helium at the temperature of 500 K and changed the temperature of the graphene from 1000 K to 1500 K and 2000 K to understand the effect of graphene temperature. Based on the significance of the zenith angle on the surface scattering, we also explored systems with ten different zenith angles and the azimuthal angle at 0° and 90°. Figure 4a shows that the TAC was still very sensitive to the zenith angle and insensitive to the azimuthal angle in the cases of different graphene temperatures. TAC increased with larger zenith angles. However, the increase in TAC was restrained by higher graphene temperatures. When the zenith angle was small, and the normal velocity component of helium dominated, systems with higher graphene temperatures had larger TACs. When the zenith angle was large and the in-plane velocity component prevailed, systems with graphene of lower temperatures had larger TACs. TAC values changed slightly when the zenith angle was 40° and 50°. Given various graphene temperatures, the trend for NNTAC was the same as that for TAC, while NTTAC had the opposite trend, as shown in Figure 4b,c. The normal kinetic energy transfer dominated in all cases of different graphene temperatures. Figure 4e,f shows that the RNTAC and RTTAC with different temperatures of graphene were consistent with NNTAC and NTTAC, respectively, except for a larger fluctuation at lower zenith angles for NNTAC and higher zenith angles for NTTAC. The zenith angle effect became insensitive with higher graphene temperatures. In all, the TACs and their components became less sensitive to zenith angle when increasing the graphene temperature.

The zenith angle of the scattered helium (*θ_o_*) shown in Figure 4d remained nonlinear relative to the zenith angle of the incident helium. As the graphene temperature increased, the nonlinearity reduced. When *θ_in_* was larger than 30°, the zenith angle *θ_o_* became smaller with higher graphene temperatures. This change increased as *θ_in_* increased. In addition, the change in *θ_o_* with graphene temperature was much lower than that in TAC and TAC components, and changes at a high *θ_in_* were larger than those at a low *θ_in_*. In the cases of the three temperatures, the *θ_in_* of specular reflection was between 30° and 40° and became smaller with higher graphene temperatures. The linear region of *θ_in_* was between 30° and 60°, and the slope became smaller with higher graphene temperatures.

Higher graphene temperatures resulted in a lower bounce number and slightly lower interaction time of helium with graphene, as shown in Figure 4g,h. The change in the bounce number became more prominent at a high zenith angle of incident helium with a higher graphene temperature. For instance, the bounce number of helium with an incident azimuth of 0° and zenith of 90° reduced from 1.798 to 1.573 and 1.428 as the temperature of the graphene increased from 1000 K to 1500 K and 2000 K, respectively. Furthermore, a large bounce number could not guarantee a high TAC. When *θ_in_* was larger than 40°, and the in-plane velocity of helium was larger than the normal velocity, a higher bounce number corresponded to a larger TAC. However, when *θ_in_* was small and the normal velocity prevailed, a higher bounce number corresponded to a lower TAC. The bounce number of helium with incident azimuth of 0° and zenith of 90° reduced from 1.200 to 1.174 and 1.158 as the graphene temperature increased. Figure 4h shows that the interaction time was consistent with the bounce number. A higher graphene temperature led to a slightly shorter interaction time. These results show that energy transfer between helium and graphene is very complicated, and the common wisdom that energy transfer is positively correlated with the bounce number was not valid in this study.

To further understand the effect of surface temperature, we plotted the relative potential energy of the system by fixing the in-plane position of helium at the location of point six and changing the normal distance of helium from the graphene. Figure 4i indicates that a higher graphene temperature leads to a more significant fluctuation in relative potential energy and the mean value of the potential energy was larger with a higher graphene temperature. In detail, when helium was 2.6 Å away from the graphene surface, the relative potential energy was 0.103 eV, 0.070 eV, and 0.034 eV, with fluctuations of 0.195 eV, 0.138 eV, and 0.067 eV, when the temperature was 2000 K, 1500 K, and 1000 K, respectively. In the dynamic run, a higher graphene temperature led to stronger vibration of the graphene surface and larger variation in the distances between helium and graphene and the ones between the adjacent carbon atoms in the graphene. Since the relation between potential energy and the atomic distance was nonlinear in this range, a larger fluctuation and mean value of the relative potential was observed, and the importance of the zenith angle effect was reduced.

### 3.5. Effect of Helium Speed

**Figure 5:**
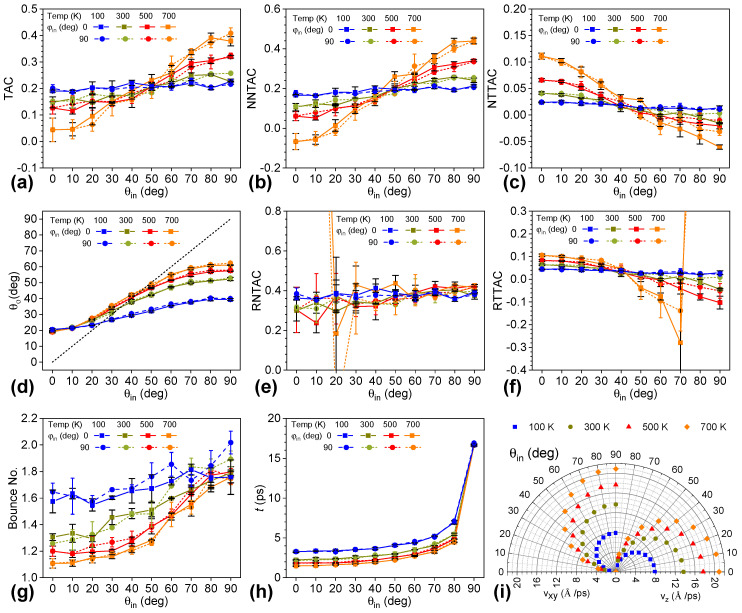
(**a**) TAC, (**b**) NNTAC, (**c**) NTTAC, (**d**) zenith angle of the scattered helium, (**e**) RNTAC, (**f**) RTTAC, (**g**) bounce number of helium, and (**h**) total collision time versus zenith angle of the incident helium with the azimuthal angle at 0° and 90°, respectively, and the speed of helium is set at the temperatures of 100 K, 300 K, 500 K, and 700 K. The graphene temperature was 1000 K. In (**e**,**f**), the curves for 700 K were out of scope at one end. (**i**) Polar plot of in-plane (v_xy_, left panel) and normal (v_z_, right panel) components of incident helium with the zenith angle of the incident helium.

To understand how the speed of helium affects energy transfer, we fixed the temperature of the graphene at 1000 K and initialized incident velocities of helium corresponding to 100 K, 300 K, 500 K, and 700 K. Figure 5a shows that the dependency of the TAC on the zenith angle of incident helium reduced with decreasing helium temperatures. In the cases of helium at 300 K, 500 K, and 700 K, systems with larger *θ_in_* showed higher TACs, but the slope of TAC relative to *θ_in_* became smaller from 700 K to 300 K. In the case of 100 K, the TACs of the systems were insensitive to the *θ_in_*. When the incident angle was smaller than 50°, we found that TACs decreased with higher helium temperatures. In contrast, TACs increased with higher incident speeds of helium when *θ_in_* was larger than 50°. Figure 5b,c shows that TAC had the same trend with various zenith angles as NNTAC but the opposite trend to NTTAC. Generally, the energy transfer via the normal velocity component still outperformed that via the in-plane velocity component. However, in the case of 700 K and a *θ_in_* of 0°, the NNTAC was smaller than the NTTAC, where the in-plane energy transfer dominated. In the case of 100 K, both NNTAC and NTTAC were not dependent on *θ_in_*. RNTAC and RTTAC showed a similar pattern with helium temperature and *θ_in_* as NNTAC and NTTAC, respectively, as shown in Figure S4. The fluctuation in RNTAC at low *θ_in_* and RTTAC at high *θ_in_* was larger with higher incident speeds of helium. It is worth noting that both the in-plane and normal velocity components changed at the same proportion when we changed the speed of helium with a fixed *θ_in_*.

The nonlinearity between *θ_o_* and *θ_in_* remained for all cases with helium temperatures from 100 K to 700 K, as shown in Figure 5d. For systems with lower helium temperatures, the enhancement in *θ_o_* with a larger *θ_in_* would be suppressed as *θ_in_* was greater than 20°, and the slope of the linear segment of the *θ_o_* against *θ_in_* curve decreased. In this way, the specular reflectance angle of the incident helium was in the range of 20°~30°, 30°~40°, 40°~50°, and 40°~50° for helium temperatures of 100 K, 300 K, 500 K, and 700 K, respectively. When the incident helium had no normal velocity component and in-plane velocity along the *x*-axis, the corresponding *θ_o_* was 39.36°, 52.32°, 57.47°, and 61.09°, respectively. In the case of 100 K, the nonlinearity was significantly reduced. In this way, reducing the temperature of helium could suppress the effect of the zenith angle of incident helium.

Different from the above-mentioned results, Figure 5g,h depicts that the bounce number and the interaction time of helium were susceptible to the speed of incident helium. Moreover, the influence of the azimuth on the bounce number of helium was enlarged with a lower incident helium speed. Especially at a low *θ_in_*, the bounce number increased drastically with a lower incident helium speed. For instance, the values were 1.58, 1.32, 1.20, and 1.11 with increasing helium speed and a *θ_in_* of 0°. When the *θ_in_* was smaller than 50°, the bounce number was consistent with the TAC. A larger *θ_in_* reduced the difference in the normal velocity component of the helium atom, shown in Figure 5i, and the difference in the bounce number. When *θ_in_* was larger than 50°, the larger bounce number caused by the lower speed of helium corresponded to a lower TAC. Furthermore, the lower incident speed of helium significantly increased the interaction time due to the lower normal velocity of helium and higher bounce number. All these results show the significance of the incident speed of helium on scattering behavior.

### 3.6. Conversion of Angle-Resolved TAC to TAC with Gas Molecules from Hemispherical Space

**Figure 6:**
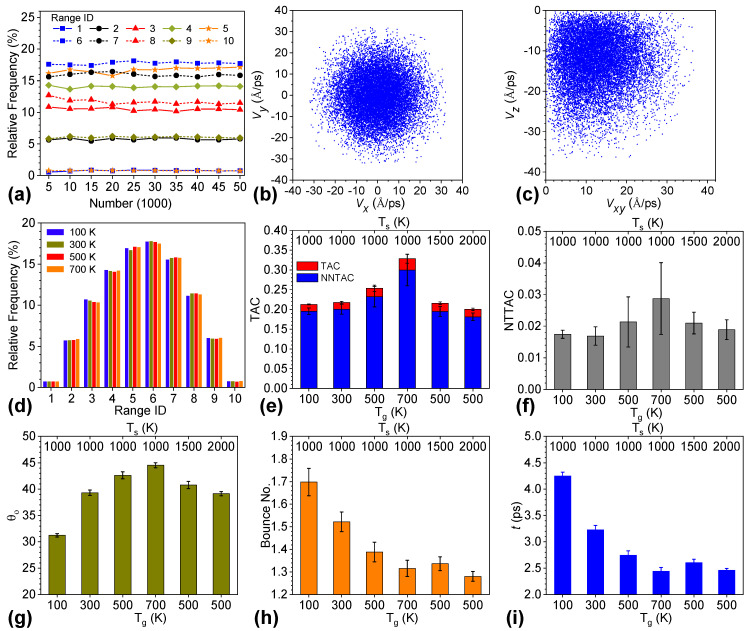
(**a**) Convergence analysis of the relative frequency of the zenith angle of incident helium. Range ID of 1 to 10 represents zenith ranges of [0°,5°], [5°,15°], … [85°,90°]. For each middle range ID, the interval width was 10°. (**b**) Scatterplot of in-plane velocity components of sampled incident helium. (**c**) Scatterplot of normal and in-plane velocity components of sampled incident helium. (**d**) Converged relative frequency in various ranges of θin for helium with different incident speeds. (**e**) TAC and NNTAC and (**f**) NTTAC for systems with various graphene temperatures denoted by top tick labels and helium incident speeds shown by bottom tick labels. (**g**) Zenith angle of scattered helium, (**h**) bounce number, and (**i**) interaction time of helium for various systems.

Since the scattering process of helium on graphene is insensitive to the azimuth, we obtained the zenith-angle-dependent TAC, NNTAC, NTTAC, bounce number, and interaction time of helium on graphene. Then, we could describe the TAC considering all molecules from the hemispherical space above the graphene surface. The relative frequencies in ten different ranges of *θ_in_* converged when the number of samples was above 25,000 in the Monte Carlo simulations, as shown in Figure 6a. The range ID from one to ten represents the zenith ranges of [0°,5°], [5°,15°], … [85°,90°]; i.e., for each middle range ID, the interval width was 10°. Figure 6b,c shows scatterplots of the in-plane and normal velocity distributions. For a clearer scatterplot, we present a distribution with 10,000 samples. The in-plane velocity plot of *V*_x_ against *V*_y_ represents a uniform distribution along the azimuth and becomes sparser along the radial direction from the zenith point. However, this does not mean that there would be a higher distribution with lower helium speed and zenith angle. The relative frequencies of *V*_x_, *V*_y_, *V*_z_, and *V* are shown in Figure S5. *V*_x_ and *V*_y_ followed a normal distribution, while *V*_z_ and *V* were in a right-skewed distribution. At a higher incident helium speed, *V*_x_ and *V*_y_ were narrower around the mean value, but *V*_z_ and *V* were right-shifted. Figure 6c depicts that the distribution with low and high zenith angles was low. Instead, the most probable distribution of the zenith angle of incident helium was between 45° and 55°. The expected *θ_in_* was 45.55° according to the given relative frequency in each range of the zenith angle shown in Figure 6d, which was used to weight the zenith angle-resolved TAC for the TAC of helium molecules in the hemispherical space.

Figure 6e presents the TAC and NNTAC for systems with various graphene temperatures and speeds of incident helium denoted by top and bottom tick labels, respectively. The blue bars for NNTAC are on top of the red bars for TAC, and indicate that the NNTAC contributed more than 90% to the TAC for all cases. Interestingly, TAC, NNTAC, and NTTAC (see Figure 6f) exhibited a similar dependence on the graphene temperature and the helium incident speed. All of them increased with increasing helium incident speed from 100 K to 700 K, except for a minor drop in NTTAC from 0.0174 to 0.0169 as *T*_g_ increased from 100 K to 300 K. In terms of the zenith-angle-resolved counterparts, these three parameters had various relationships with zenith angle and helium temperature. They displayed the opposite dependence on helium temperature at low and high zenith angles. However, they were balanced and resulted in the trend observed for the integrated TAC. Moreover, all three parameters decreased with higher graphene temperatures. In this regard, we could improve the TAC via lower-temperature graphene and higher-temperature helium gas.

Figure 6g–i display the corresponding zenith angle of scattered helium, bounce number, and interaction time of helium with graphene. Given a graphene temperature of 1000 K, *θ_o_* increased from 31.23° to 39.32°, 42.62°, and 44.55° as helium temperature increased from 100 K to 700 K. The higher the temperature of helium, the closer its scattering behavior approached the expected value, 45.55°, of the incident zenith angle. Furthermore, *θ_o_* decreased from 42.62° to 40.78° and 39.14° when increasing the graphene temperature from 1000 K to 1500 K and 2000 K, respectively, and fixing the temperature of helium at 500 K. These data indicate that *θ_o_* became closer to the expected value of *θ_in_* upon reducing the temperature difference between helium and graphene. Contrary to common wisdom, Figure 6h,i demonstrated that higher bounce numbers and interaction times of helium with graphene could not guarantee a higher energy transfer between helium and graphene. When the graphene temperature was fixed at 1000 K, the bounce number of helium decreased from 1.70 to 1.52, 1.39, and 1.32 for helium temperatures of 100 K, 300 K, 500 K, and 700 K, respectively. In this case, the interaction time declined, which was consistent with the trend for the bounce number. However, this trend was opposite to that for the TAC. On the other hand, the bounce number and interaction time decreased with higher graphene temperatures, which agreed very well with the trend for the TAC.

## 4. Conclusions

In this work, we investigated the energy transfer and scattering behavior of helium with graphene. We performed molecular dynamics simulations of angle-resolved scattering performance, conducted Monte Carlo simulations for the relative frequencies at various ranges of the zenith angle of the incident helium atom, and hence obtained integrated scattering performance considering all molecules from the hemispherical space. Furthermore, the effects of graphene temperature and incident helium speed corresponding to different temperatures on helium–graphene scattering behavior were elucidated.

In terms of the angle-resolved scattering of helium on graphene, scattering performance includes the thermal accommodation coefficient (TAC), the zenith angle of scattered helium, the bounce number, and the interaction time of helium with graphene. Performance was insensitive to the azimuthal angle of the incident helium but highly dependent on the zenith angle. Moreover, the nominal normal thermal accommodation coefficient (NNTAC) and nominal tangential thermal accommodation coefficient (NTTAC) were defined by decomposing the kinetic energy transfer into the in-plane and normal velocity components of helium. We found that TAC and NNTAC increased nonlinearly with larger zenith angles in the cases of incident helium at 500 K and graphene at 1000 K. In contrast, the NTTAC decreased with increasing zenith angle. However, NNTAC outweighed NTTAC, and kinetic energy transfer via the normal velocity of helium dominated the energy transfer between helium and graphene. The real normal thermal accommodation coefficient (RNTAC) and real tangential thermal accommodation coefficient (RTTAC) defined by the Maxwell Model were consistent with the NNTAC and NTTAC, respectively, except for larger fluctuations in RNTAC at a low zenith angle and RTTAC at a large zenith angle. The zenith angle of the scattered helium nonlinearly increased with a larger zenith angle of incident helium. The zenith angle of the scattered helium was linearly correlated with the zenith angle of the incident helium in the middle range from 30° to 60°. However, it deviated from a linear correlation significantly at either low or high zenith angles of incident helium. The scattered helium had the same zenith angle, approximately 44°, as the incident helium. The bounce number increased with higher zenith angles of incident helium. In the case of a constant incident helium speed and graphene temperature, it was consistent with our hypothesis that a higher bounce number corresponded to greater energy transfer between the gas and the solid surface. Helium took more time to interact with the graphene when the zenith angle of incident helium was larger. In particular, an extremely long flight time was required when the incident helium speed was parallel to the graphene surface. The effect of the zenith angle of the incident helium atom stemmed from a heterogeneous distribution of potential energy between helium and graphene, and the dynamic vibration of the graphene atoms mitigated the differences in the potential energy of the system given various in-plane locations of helium.

When the incident helium speed was fixed and the graphene temperature was enhanced, the nonlinear behavior of the angle-resolved parameters of the TAC, NNTAC, NTTAC, RNTAC, and RTTAC, the zenith angle of the scattered helium, and the bounce number of helium were suppressed. The dependence of these parameters on the zenith angle of incident helium was different at low and high zenith angles of incident helium. For instance, TAC, NNTAC, and RNTAC increased with higher graphene temperatures at low zenith angles of incident helium, but decreased at higher zenith angles of incident helium. However, the NTTAC and RTTAC had opposite trends. The reduction in the zenith angle of scattered helium and the bounce number at higher graphene temperatures was much more severe at a higher zenith angle of the incident helium. The interaction time of helium decreased slightly with a higher graphene temperature. Relative potential analysis of the system showed a bigger potential-energy change at higher graphene temperatures due to a larger vibration among carbon atoms in the graphene, which resulted in a change in the scattering performance.

When the graphene temperature was fixed and the incident helium speed was changed, the nonlinear behavior of the angle-resolved parameters of the TAC, NNTAC, NTTAC, RNTAC, and RTTAC, the zenith angle of the scattered helium, and the bounce number of helium could be suppressed by reducing the incident helium speed. The phenomena observed when increasing the graphene temperature regarding TAC, NNTAC, TTAC, RNTAC, RTTAC, bounce number and interaction time could also be achieved by decreasing the incident helium speed. Significantly, the TAC, NTAC, NNTAC, RNTAC, and RTTAC were insensitive to the zenith angle of incident helium at 100 K. In this section, we found that a large bounce number of helium and longer interaction time between helium and graphene could not guarantee a higher TAC and higher energy transfer.

Integrated scattering performance was obtained through weighting the values of the parameters with various zenith angles of the incident helium via the relative frequencies of the zenith angles by Monte Carlo simulation, and showed the scattering performance of helium on graphene considering all molecules in the hemispherical space. The Monte Carlo simulations considered the fast atom effect of helium atoms. The result showed that the zenith angle of incident helium followed a normal distribution with an expected value of 45.55°. The integrated TAC, NNTAC, NTTAC, and bounce number of helium increased with higher incident helium speeds and decreased with higher graphene temperatures. Significantly, the NNTAC accounted for more than 90% of the TAC. The integrated bounce number of helium was consistent with the interaction time of helium with graphene. The intuition that larger bounce numbers and longer interaction times correspond to higher transfers of energy is only valid when disregarding the change in incident helium speed. As the incident helium speed changes, larger bounce numbers and longer interaction times could show lower TAC values. In this study, we provided a clear description of the scattering performance of helium on high-temperature graphene surfaces in both angle-resolved and integrated aspects. These findings can provide a better fundamental understanding and a new way, such as through incident angles, to tune the scattering behavior of gas molecules on 2D materials.

## Data Availability

The data presented in this study are available on request from the corresponding author L.Z.

## Supplementary Materials

The following supporting information can be downloaded at: https://www.mdpi.com/article/10.3390/nano12162855/s1. Figure S1: Fluctuations of carbon atoms and accumulative average TACs for the production MD system and the system with enlarged graphene area; Figure S2: Convergence analysis of TAC and its components; Figure S3: Contour plot of potential energy in the y–z plane; Figure S4: RNTAC and RTTAC versus zenith angle for various incident helium speeds; Figure S5: Distributions of the speed and velocity components of helium at various temperatures.
