# Supplementary material for: Thermal Energy Transfer between Helium Gas and Graphene Surface According to Molecular Dynamics Simulations and the Monte Carlo Method
Source: Nanomaterials (Basel). 2022 Aug 18;12(16):2855. doi: 10.3390/nano12162855 (PMC9416252; doi:10.3390/nano12162855)
Supplement: Supplementary file 1 [file nanomaterials-12-02855-s001.zip › nanomaterials-1816834-supplementary.pdf]

Thermal Energy Transfer between Helium Gas and Graphene Surface According to Molecular Dynamics Simulations and the Monte Carlo Method

Lin Zhang [1,*] and Heng Ban [2]

[1] Department of Engineering Mechanics, School of Civil Engineering, Shandong University, Jinan 250061, China
[2] Department of Mechanical Engineering and Materials Science, University of Pittsburgh, Pittsburgh, PA 15261, USA; heng.ban@pitt.edu
* Correspondence: linzhang1629@email.sdu.edu.cn

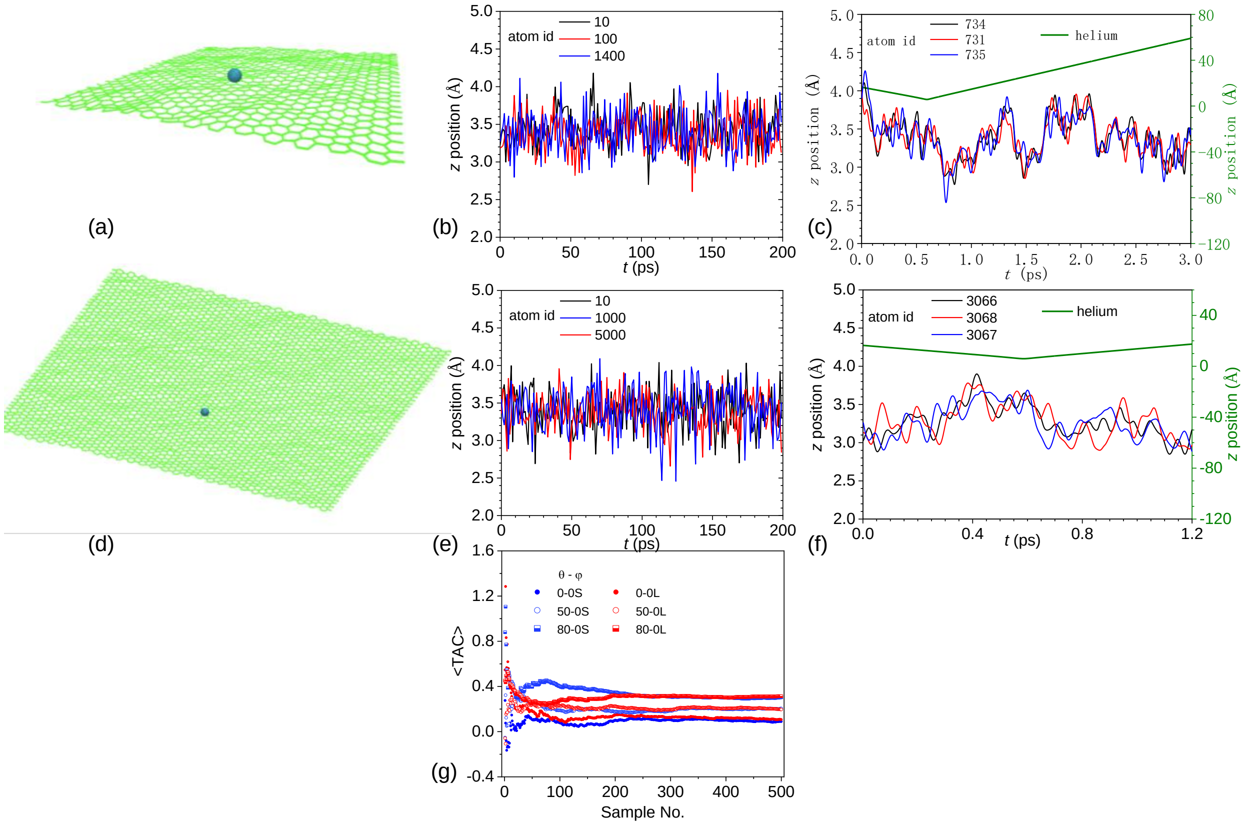

Figure S1. The fluctuations of carbon atoms and accumulative average TACs for the production MD system (**a–c**) and system with enlarged graphene (**d–f**). The schematic plot of the graphene system for the production run is shown in (**a**), and a system with graphene doubled in size in both x and y directions is shown in (**d**). The fluctuations of atoms during the NVE simulation are shown in (**b**) and (**e**) for the original and enlarged systems, respectively. Three carbon atoms were randomly selected with atom IDs of 10, 100, and 1400 for the former and 10, 1000, and 5000 for the latter. The fluctuations during the scattering process are shown in **Error! Reference source not found.**(**c**) and (**f**), respectively. The three closest atoms were selected when the helium atom reached the minimum position in the z direction during the scattering process. The cyan lines are used to indicate the timeline of the scattering process by the z position of the helium atom, where the tick labels and title for the helium atom are in cyan on the right. (**g**) the accumulative average TACs for both systems converge with the sampling number. The blue curves indicate the systems

with the zenith incident angles of 0°, 50°, and 80°, while the red ones are for the counterparts of the enlarged graphene system. The symbols "S" and "L" denote the size of the graphene in the system.

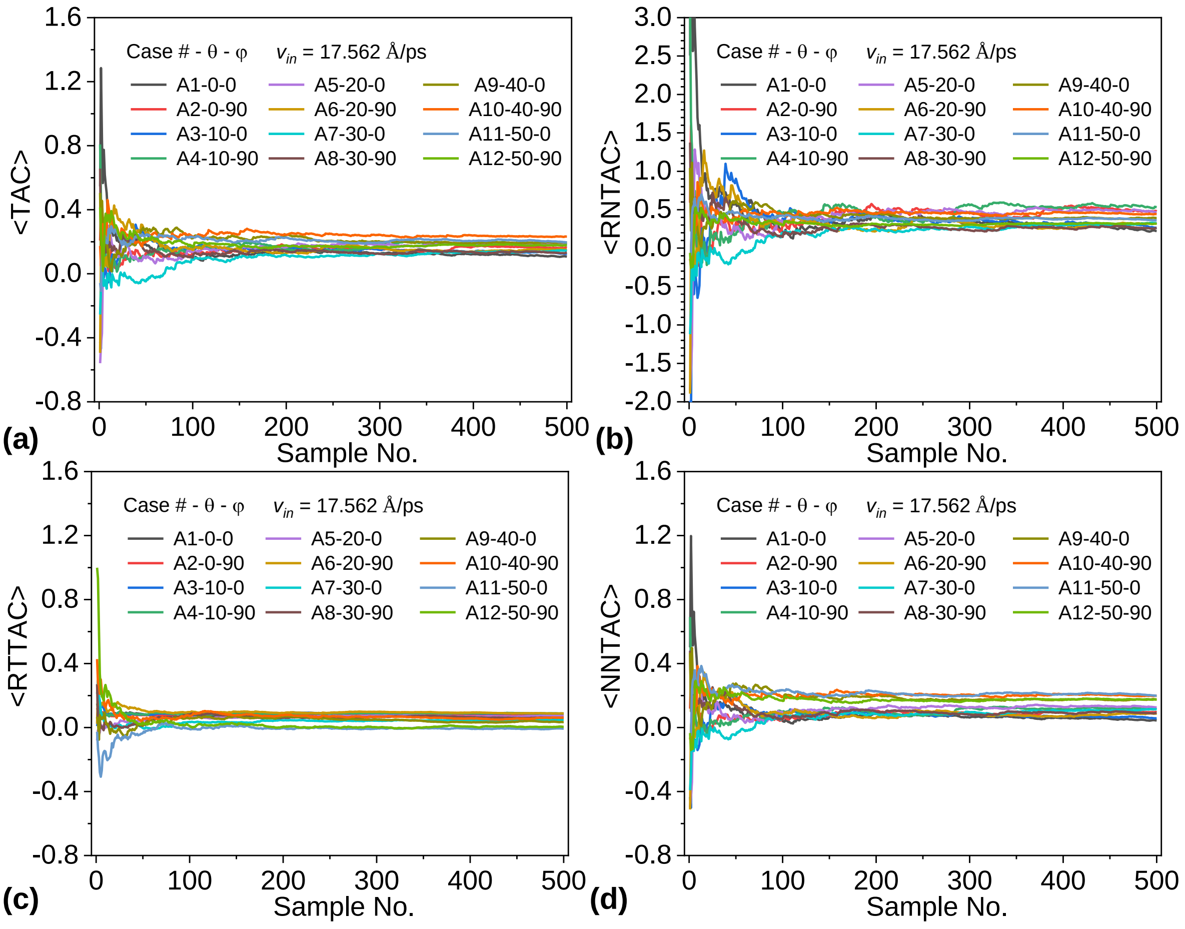

Figure S2. Convergence analyses of (**a**) thermal accommodation coefficient (TAC), (**b**) real normal thermal accommodation coefficient (RNTAC), (**c**) real tangential thermal accommodation coefficient (RTTAC) and (**d**) nominal normal thermal accommodation coefficient (NNTAC) with the number of samples. <·> denotes the cumulative average of the specified number of samples. In all cases, the speed of the incident helium atom is 17.652 Å/ps, which corresponds to the root mean square velocity of helium at 500 K. θ and φ denote the zenith angle and the azimuth angle of incident helium in degrees. We can obtain that TACs converge with 500 simulation samples.

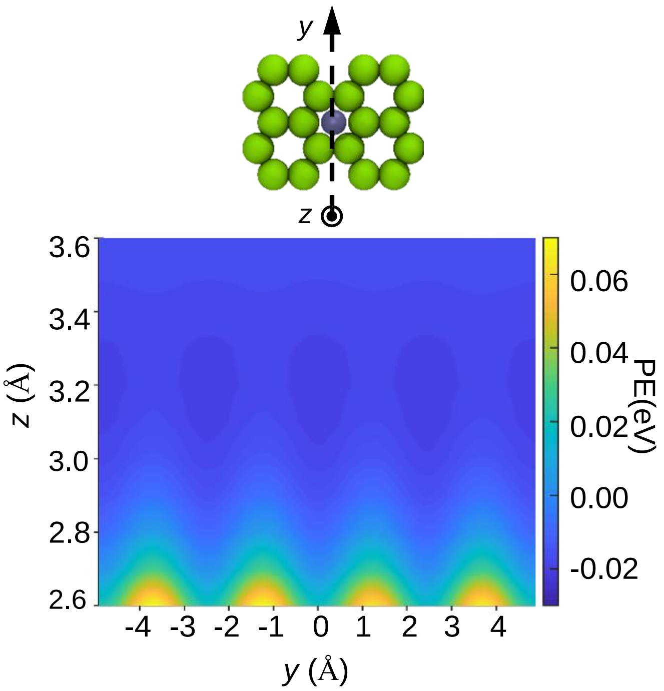

Figure S3. Contour plot of the potential energy of helium atom in the denoted y-z plane. The reference potential energy was set when the helium atom was 20 Å above the graphene top surface.

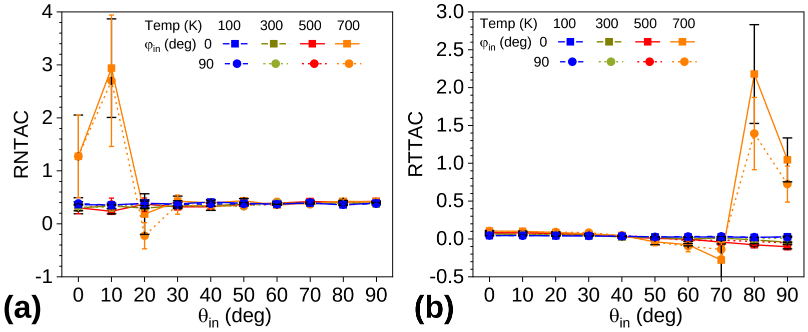

Figure S4. (**a**) RNTAC and (**b**) RTTAC versus the zenith angle of the incident helium with the azimuthal angle at 0˚ and 90˚, respectively. The speed of helium is set at the temperature of 100K, 300 K, 500 K, and 700 K. The temperature of graphene is 1000 K.

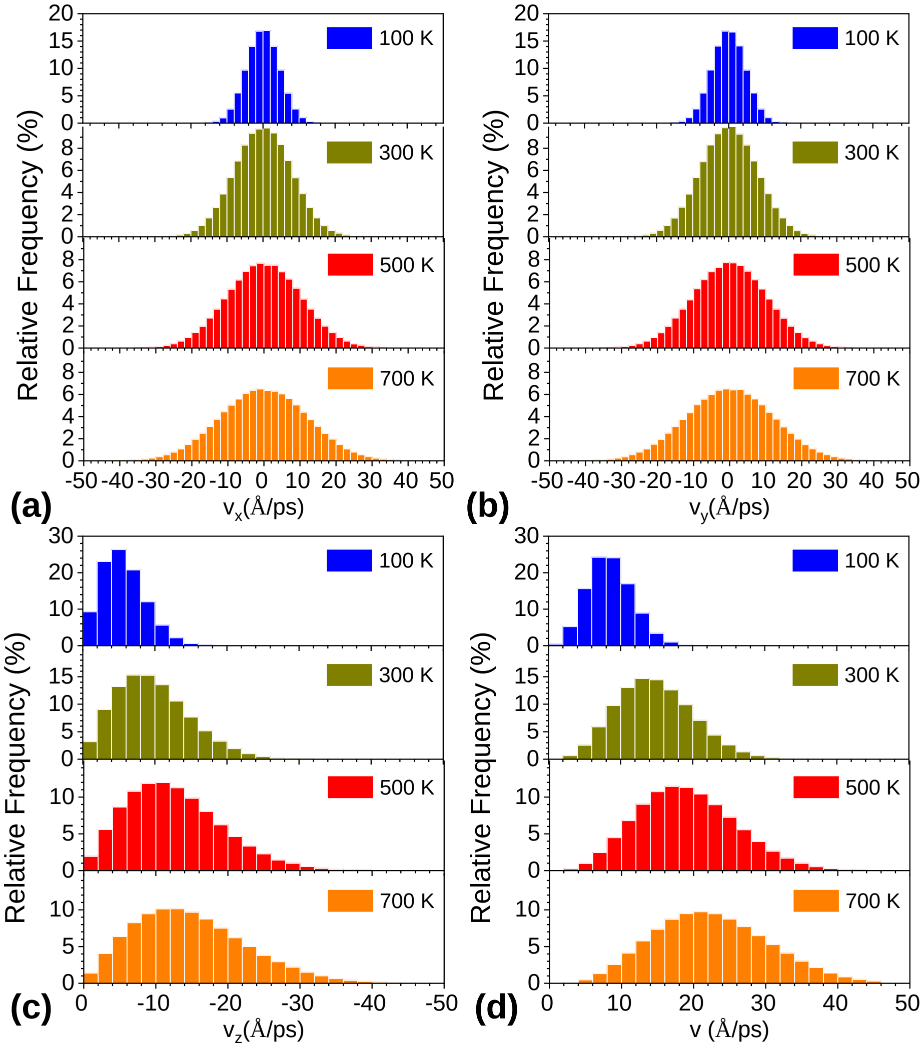

Figure S5. The relative frequencies of (**a**) V_x, (**b**) V_y, (**c**) V_z, and (**d**) V for the incident helium at the temperatures of 100 K, 300 K, 500 K, and 700 K.
